# Field performance evaluation of the GenBody ConfiSign HIV self-test for blood-based HIV screening in Uganda

**DOI:** 10.1371/journal.pone.0361282

**Published:** 2026-10-27

**Authors:** Judith Kyokushaba, Dennis Mujuni, Viola Kasone, Aida Namakula, Caroline Makoha, Flaviano Mugalula, Christopher Okiira, Wilson Nyegenye, Christine Katusiime, Robert Mutumba, Susan Nabadda

**Affiliations:** 1 National Health Laboratory and Diagnostic Services, Ministry of Health, Kampala, Uganda; 2 Makerere University, College of Health Sciences, Kampala, Uganda; 3 Ministry of Health, AIDS Control Program, Kampala, Uganda; Kwame Nkrumah University of Science and Technology, GHANA

## Abstract

HIV self-testing (HIVST) is a crucial strategy to reach the UNAIDS 95-95-95 targets by increasing testing coverage in high-burden areas like Uganda. The ConfiSign HIV Self-Test, a blood-based, direct-sampling, vertical flow, single-use rapid test, demonstrated perfect sensitivity and specificity during laboratory validation. This study assessed its performance and operational characteristics in the field compared to the Wondfo HIV Self-Test, which served as the standard of care. A cross-sectional mixed-methods study was conducted from 09 April 2025 to 30 May 2025 at 15 health facilities in Uganda. Participants (n = 2,434, aged ≥14 years) performed self-testing with ConfiSign and Wondfo (standard of care) under observation. Diagnostic accuracy was calculated against Wondfo. Usability, acceptability, and feasibility were evaluated via questionnaires (n = 798 for usability/acceptability; n = 90 for feasibility). Discordant results were resolved using ELISA and PCR. Participants were predominantly female (61.7%) and aged 18−29 (51.8%). ConfiSign positivity was 6.2% versus 6.0% for Wondfo. Sensitivity was 100%, specificity 99.8% (95% CI: 99.53–99.93). Three discordant results were resolved as negative via PCR and ELISA. Usability was high, with 94.7% of users finding components easy to handle and 83.3% collecting samples without difficulty. Acceptability was strong, as 98.0% would reuse the test and 99.0% would recommend it. Feasibility assessments across operational, usability, performance, economic, and acceptability domains yielded positive scores, supporting integration into Uganda’s HIV testing framework. These findings confirm the kit’s diagnostic accuracy and ease of use in field settings, supporting its adoption for HIVST in Uganda. We suggest improvements such as instructions in the local language and the inclusion of additional gauze for better acceptance.

## Introduction

The HIV pandemic continues to disproportionately affect sub-Saharan Africa [1], where 25.6 million of the global 39 million people living with HIV (PLHIV) resided in 2022 [2]. Uganda has progressed toward the UNAIDS 95-95-95 goals, with 89% of PLHIV aware of their status by 2019 [3]. However, challenges like stigma, limited access to testing services, and missed testing opportunities in healthcare persist [4–6].

HIVST offers a discreet alternative to facility-based testing, potentially reaching underserved groups such as men, key populations, and youth. The World Health Organization (WHO) recommends expanding HIVST for general populations in high-burden areas and for PrEP [6,7]. Rapid HIV assays have advanced, but performance varies by context, necessitating local evaluations [8–10].

Since 2016, Uganda’s Ministry of Health (MOH) has validated kits like OraQuick, SURE CHECK, INSTI, Wondfo, and CHECKNOW. Despite the growing availability of HIV self-testing (HIVST) kits, evidence on newer options like the ConfiSign remains scarce in Uganda. This gap in data hinders efforts to refine testing tools, achieve WHO prequalification, and expand national adoption to overcome barriers such as stigma, limited access to testing centers, and privacy concerns.

The GenBody ConfiSign HIV SelfTest (ST) (GenBody Inc., Cheonan, Republic of Korea) is a rapid in vitro qualitative Vertical flow test for the detection of antibodies to Human Immunodeficiency Virus (HIV) using a nitrocellulose membrane that is immobilized with HIV-1 antigens (gp41 + gp120) and HIV-2 antigen(gp36) in the test line. The recombinant HIV antigens (gp41 + gp120 + gp36) are conjugated to colloidal gold particles and placed on a polyester or glass pad. The test uses a wide variety of samples, such as human whole blood, finger stick blood, or plasma (I-208-EN-R0.1-240607(ConfiSign HIV Self-Test)-Uganda (1).pdf).

The sensitivity and specificity of the GenBody ConfiSign HIV Self-Test, compared to the established HIV blood-testing algorithm in Uganda, are 100% and 100%, respectively, as reported in the 2024 UVRI/MOH in-house laboratory validation report [11]. In order to further evaluate this and address the aforementioned gaps, our study evaluated the field performance of the ConfiSign HIV Self-Test compared to the Wondfo HIV Self-Test. We evaluated diagnostic accuracy by measuring sensitivity, specificity, positive predictive value (PPV), and negative predictive value (NPV). We also assessed the kit’s feasibility, acceptability, and usability across various health facilities to guide its potential integration into Uganda’s HIV testing strategy.

## Methods

### Study design

This cross-sectional mixed-methods study combined quantitative and qualitative approaches. Quantitative data captured diagnostic accuracy via registers, while qualitative data assessed usability, acceptability, and feasibility through questionnaires. The study was conducted from 09 April 2025 to 30 May 2025 at 15 health facilities selected for high HIV prevalence, patient volume, and prior evaluation experience. Sites spanned outpatient departments (OPDs) and laboratories across regional referral hospitals, general hospitals, and lower-level health centers IVs, representing diverse regions (Table 1).

**Table 1:** Sites Selected for the Study.

Region	Facility Name
Southcentral	Masaka Regional Referral Hospital; Mityana General Hospital
Central	Entebbe Regional Referral Hospital; Wakiso HC IV; Mpigi HC IV
East Central	Bugembe HC IV; Jinja Regional Referral Hospital
North Central	Mukono General Hospital; Luwero General Hospital
Western	Mbarara Municipal HCIV; Kibito HC IV; Fort Portal Regional Referral Hospital; Kabale Regional Referral Hospital
West Nile	Yumbe Regional Referral Hospital
North	Lira Regional Referral Hospital

### Participants and eligibility

A total of 2,434 participants were enrolled across 15 health facilities. To evaluate the feasibility of the ConfiSign HIV Self-Test in a diverse population, we included individuals aged 14 years and older who were seeking HIV testing and provided informed consent.

We excluded pregnant women attending antenatal care due to their unique physiological needs and existing specialized protocols; individuals with visual or cognitive impairments that would hinder independent use of the test; and persons with known HIV-positive status, as this would confound assessment of the test in undiagnosed individuals. These exclusions ensured participant safety and study validity.

These criteria ensured that the study focused on the feasibility and acceptability of self-testing among individuals most likely to benefit from independent HIV test use.

### Procedures

#### Training and study implementation workflows.

At each participating facility, leaders nominated two health workers, one from the Outpatient Department (OPD) and one from the laboratory. These individuals underwent training at the Central Public Health Laboratory (CPHL). The training covered the following content (Table 2).

**Table 2:** Components Involved in the Training.

Component	Description
Kit Usage	Hands-on with ConfiSign and Wondfo
National Algorithm	Refresher on Determine, Stat-Pak, Bioline
Data Tools	Registers, questionnaires, checklists
Ethics	Consent, confidentiality, linkage
Supervision	Reporting, reviews

This approach ensured that both clinical and laboratory perspectives were represented, while strengthening capacity for consistent implementation across all sites.

Following the central level training, on-site facility orientations were conducted, commodities were distributed, and participant recruitment was completed within 52 days (09 April 2025 to 30 May 2025). Eligible clients provided informed consent and performed HIV self-testing under direct observation.

#### Participants used the ConfiSign HIV self.

After completing the test, clients filled out usability and acceptability questionnaires in English. Final results were interpreted in line with national HIV self-testing (HIVST) guidelines:

- Negative results were confirmed using Wondfo.
- Positive results were verified through the national algorithm (Determine, Stat‑Pak, Bioline).
- Discordant results were resolved at the Central Public Health Laboratory (CPHL) using 4^th^-generation ELISA and PCR assays.

Health workers submitted biweekly data reports and participated in regular review meetings. Supervision visits were conducted at the start, midpoint, and conclusion of the study to ensure fidelity to protocols and maintain quality assurance.

### Data collection and management

#### Data collection.

We designed a comprehensive data collection strategy that captured both quantitative and qualitative insights from participants across 15 health facilities in Uganda to thoroughly assess the ConfiSign HIV Self-Test’s performance and operational characteristics. This approach aimed to ensure robust evidence on diagnostic accuracy, usability, acceptability, and feasibility in real-world settings. Quantitative data collection centered on a custom-designed study register, the “ConfiSign Register,” which documented key variables for each of the 2,434 participants enrolled between 09 April 2025 to 30 May 2025. These variables included test date, unique participant identifier, age, sex, and results from the ConfiSign test, the Wondfo reference test, and the national HIV testing algorithm. Two trained research assistants per facility, including one from the outpatient department and one from the laboratory, both oversaw this process. This ensured consistent documentation while adhering to standardized protocols.

Qualitative data provided deeper insights into user experiences, drawing from 798 participants who completed acceptability and usability questionnaires in English, guided by trained health workers. These questionnaires explored practical aspects, such as ease of sample collection and result interpretation, as well as subjective perceptions, including confidence in the test and willingness to recommend it. Health care workers also completed the observer checklists for each participant, recording adherence to test procedures and any challenges, such as difficulties with the fingerstick device. These diverse data sources enriched our understanding of the test’s practical utility.

#### Data management.

We utilized EpiData software to manage both quantitative and qualitative data, selected for its reliability in handling large datasets and facilitating verification. Research assistants at each site entered data into EpiData, with entries meticulously cross-checked by a study coordinator to confirm accuracy and completeness. We assigned unique identifiers to each participant, thereby excluding identifiable information from electronic records and safeguarding participant confidentiality. Physical documents, including registers and questionnaires, were securely stored under lock and key at each facility, aligning with the Ministry of Health’s ethical guidelines. This rigorous management process minimized errors and protected participant privacy, ensuring the data’s reliability for subsequent analysis.

#### Data analysis.

Analysis leveraged STATA version 17, chosen for its robust capabilities in handling the complex statistical requirements of this study. To evaluate the ConfiSign’s diagnostic performance, we calculated sensitivity, specificity, positive predictive value (PPV), and negative predictive value (NPV), with 95% confidence intervals, using the Wondfo HIV Self-Test as the gold standard. This approach quantified the test’s ability to accurately identify HIV-positive and HIV-negative individuals in field conditions. Multinomial logistic regression explored predictors of usability challenges, such as education level and procedural ease, revealing that higher education significantly reduced sample collection difficulties. Chi-square tests assessed associations between user confusion and result interpretation accuracy, with a significant finding (p = 0.032) indicating that confusion impacted line identification. Logistic regression further identified procedural errors as a predictor of misinterpretation.

Feasibility analysis involved computing composite scores for five domains: operational, usability, performance, economic, and acceptability, as derived from Likert-scale responses in the feasibility questionnaire. These scores provided a holistic view of the test’s integration potential, with performance scores about the Mean, and Standard Deviation (SD) underscoring user trust in result accuracy. Integrating quantitative metrics with qualitative narratives, such as participant calls for local language instructions, allowed a comprehensive assessment of ConfiSign’s suitability for Uganda’s HIV testing landscape.

### Ethical considerations

This study received ethical approval from the Uganda National Health Laboratory Services Research Ethics Committee (REC) (Reference number: UNHL-2025–102). Approval was granted at the 64th convened REC meeting held on 27 February 2025, with an approved study period from 07 March 2025 to 07 March 2026. The research protocol was subsequently registered with and approved by the Uganda National Council for Science and Technology (UNCST) on 02 April 2025 (Registration number: HS5760ES), with approval valid from 02 April 2025 to 02 April 2026.

Participant recruitment was conducted from 09 April 2025 to 30 May 2025 at 15 health facilities in Uganda. All participants were prospectively enrolled and provided informed consent before participation. Written informed consent was obtained from all participants aged 18 years and older. For adolescents aged 14–17 years, written assent was obtained in addition to written consent from a parent or legal guardian, in accordance with national ethical guidelines and REC-approved procedures. Only REC-approved consent and assent forms were used for participant enrollment, and all signed consent documentation was retained as required. Participants were informed of their right to withdraw at any time without penalty, and all processes prioritized voluntary participation. This approach not only aligned with REC and UNCST requirements but also addressed cultural sensitivities through multilingual materials, fostering trust and inclusivity in diverse Ugandan communities. All study procedures were implemented in accordance with the approved protocol and under the oversight of the REC and UNCST.

The study adhered to MOH standard operating procedures for HIV testing and infection prevention, with positive results triggering immediate linkage to care and no other direct benefits promised. Adverse events, unanticipated problems, or protocol/consent amendments were reported promptly to the REC, UNCST, and National Drug Authority as applicable, with annual progress reports sustaining REC continuation approval. Participant confidentiality was maintained through unique identifiers, exclusion of personal information from electronic data, and secure physical record storage, subject to REC/UNCST audit to minimize stigma or discrimination risk.

## Results

### Participant demographics

A total of 2,434 participants were recruited for the evaluation across the 15 health facilities between May and June 2025. The demographic profile of the study population shows that the evaluation largely reached young adults, with the majority of participants aged 18–29 years (51.8%), followed by those aged >30 years (44.9%) and <17 years (3.2%). Females constituted the larger proportion of participants (61.7%), while male were 38%. Table 6 below summarizes the participant demographic characteristics beyond age and sex with 798 participants who participated in the qualitative assessments after undergoing the testing.

### Positivity rates

HIV positivity rates were comparable between ConfiSign (6.2%) and Wondfo (6.0%), with similar patterns across site categories (Table 3).

**Table 3:** HIV Positivity Rates by Site Category.

Site Category	Confisign Positivity (%)	Wondfo Positivity (%)	Sample Size
Regional Referral Hospitals	5.5	5.4	1,294
General Hospitals	8.6	8.1	418
Health IV	6.0	6.0	722
Overall	6.2	6.0	2,434

### Diagnostic accuracy

The test achieved 100% sensitivity, correctly identifying every true positive case, with a positive predictive value (PPV) of 97.3% (95% CI: 93.15–99.0). Specificity was 99.8% (95% CI: 99.53–99.93), and the negative predictive value (NPV) reached 100%. Table 4.

**Table 4:** Overall Performance of the ConfiSign Kit.

		Wondfo (Reference)		
GeneBody ConfiSign kit		Positive	Negative	Total
	Positive	147	4	**151**
	Negative	0	2,283	**2,283**
	**Total**	**147**	**2,287**	**2,434**

Sensitivity = 100%, PPV = 97.3% [95% CI: 93.15–99.0].

Specificity = 99.8% [95% CI: 99.53–99.93], NPV = 100%.

### Confirmation of the discordant results

Three samples that had discordant results between the ConfiSign, Wondfo, and the HIV National testing algorithm were referred to CPHL for discrepancy resolution using the Cobas® 6800/8800 HIV-1/HIV-2 Qualitative RNA PCR test, and the Roche cobas e 411 analyzer, a fourth-generation ELISA that detects both HIV-1 p24 antigen and antibodies. Both tests provided negative HIV results for all the samples, as seen in Table 5 below.

**Table 5:** Results for the discordant samples.

PCR results	ELISA results (Titers)
Negative	Non – reactive (0.595)
Negative	Non – reactive (0.521)
Negative	Non – reactive (0.572)

### Demographic characteristics for the qualitative feedback

Among the subset of 798 participants who provided complete qualitative feedback, respondents were predominantly female, adults aged 25–59 years, and at least primary-educated, though the sample still included adolescents, youths, older adults, and participants with no formal education. Full demographic detail is presented in Table 6.

**Table 6:** Demographic Characteristics of Participants in the Qualitative Assessment (N = 798).

Variable	Category	Frequency	Percent
Gender	Male	273	34.2
	Female	504	63.2
	Missing	21	2.6
Age group	Adolescent (14–19)	65	8.2
	Youths (20–25)	235	29.5
	Adults (25–59)	480	60.2
	Older adults (60+)	11	1.4
	Missing	7	0.9
Education level	No formal education	58	7.3
	Primary school	251	31.5
	Secondary school	296	37.1
	Higher education	174	21.8
	Missing	19	2.4

### Usability

Participants predominantly provided positive feedback on the usability of the test kit, while 94.7% finding it easy to handle the kit’s components. Additionally, 83.3% of the participants indicated easy sample management, 76.6% found it easy to follow the standard operating procedures, 83.5% reported being confident in interpreting the results, and 96.5% found the test results clear and easy to read (6). Education predicted ease (multinomial regression, p < 0.05). A substantial majority expressed willingness to use the test again (95.1%), and 95.2% would recommend it to other clients (Table 7).

**Table 7:** Distribution of Participant Responses on Usability, Comprehension, and Acceptability (N = 798).

Aspect	Very Easy/Easy (%)	Neutral (%)	Difficult/Very Difficult (%)
Handling Components	94.7	3.1	2.2
Sample Collection	83.3	8.9	7.8
Following Procedures	76.6	12.4	11.0
Result Interpretation Confidence	83.5	10.2	6.3
Result Clarity	96.5	2.1	1.4

Feedback: Challenges with lancets, suggestions for non-blood samples, C and T labels on the reading zone of the kits for easy results interpretation, and missing gauze. *“I have not found any cotton or gauze to use after pricking myself. Can this be added?”.* One participant mentioned that as one of the challenges faced during the test procedure. Another participant also noted difficulties in the results interpretation: “*I did not see the C or T labels on the new test kits.” “Can this be added?”.* Overall usability of the self‑test varied across age groups but remained consistently high. Most participants reported reading the instructions before starting the test, with the highest adherence among youths (81.2%) and the lowest among older adults (70%). Ease of understanding the instructions was generally strong, though slightly lower among adults (63.1%) compared to older adults (80%). Following test steps was easiest for youths (87.1%), while adults reported more difficulty (73.9%). Confidence in interpreting results was high across all groups, exceeding 90%, and reached 100% among older adults.

When asked about the most difficult part of the test, “reading instructions” was the most frequently cited challenge, particularly among older adults (85.7%). Despite these challenges, willingness to recommend the test was nearly universal, with over 96% of adolescents, youths, and adults, and 100% of older adults indicating they would recommend it.

Observers’ feedback: High levels of adherence to test procedures, with over 98% of participants performing each task correctly. (S1 Table), A vast majority (91.7%) made no errors; (S2 Table). Confusion affected interpretation (chi-square p = 0.032; S3 Table). A high proportion (98.3%) successfully interpreted the results; (S4 Table).

### Acceptability

High Likert scores: ease without assistance (4.2/5), no struggle (4.1), sample ease (4.0), instruction clarity (4.1), interpretation ease (4.2), trust (4.1), low anxiety (3.6) (S5 Table). 98.0% reuse, 99.0% recommend (S6 Table).

While comprehension was high overall, a subset of users struggled to interpret test instructions for use and the results themselves. One participant mentioned, *“I was not able to read and understand the instructions for use”*, “*Reading is my problem; I can’t read and understand English, and I didn’t even know the sign for negative or positive like on other test kits. Can the instructions for use of the kit be translated into the local languages?*”

### Feasibility

Five (5) major feasibility domains were assessed: operational 4.0, usability 6.1, performance 4.0, economic 4.0, and acceptability 5.0 (S7 Table). Feedback: Multilingual instructions, reduced wait time.

## Discussion

The ConfiSign HIV Self-Test emerges from this field evaluation as a robust and reliable tool with the potential to transform HIV screening in Uganda. With an estimated 1.5 million people living with HIV and testing coverage at 89%, the country remains below the UNAIDS 95‑95‑95 targets. Expanding access to self-testing through ConfiSign could help close this gap by increasing reach to populations less likely to access facility-based testing, including adolescents and men, enhancing privacy and autonomy, reducing stigma associated with HIV testing, strengthening linkage to care when paired with national algorithms and confirmatory pathways and supporting epidemic control by identifying undiagnosed cases and accelerating treatment initiation [2,3]. In our study population, young adults aged 18–29 made up the majority of participants (51.8%), a priority group for HIV testing services that stands to benefit from convenient, confidential, and facility-supported self-testing options such as ConfiSign. Field evaluation of the ConfiSign HIV Self‑Test demonstrated 100% sensitivity and 99.8% specificity (95% CI: 99.53–99.93) when benchmarked against the Wondfo assay. The kit correctly identified all true positive cases and produced only three false positives out of thousands tested, yielding a 100% negative predictive value. This performance instills strong confidence in negative outcomes and ensures reproducibility in diverse community settings.

Importantly, these findings mirror the 2024 laboratory validation conducted by UVRI and the Ministry of Health [11], reinforcing the robustness of ConfiSign across both controlled and field environments. Taken together, the evidence underscores ConfiSign’s potential as a seamless substitute for Wondfo in routine HIV screening, offering scalability across Uganda’s varied demographics and health facility tiers.

Comparisons with other evaluations reinforce this strength. Majam et al.’s 2021 South African study tested four kits, reporting sensitivities of 99.7% for BioSURE (a repackaged Chembio SURE CHECK HIVSK), 99% for INSTI, and 99.3% for OraSure, with specificities at or near 100% [12]. ConfiSign’s 100% sensitivity and 99.8% specificity align and compare favorably to kits like BioSURE (99.7%/100%) [12].

ConfiSign surpasses these in sensitivity, hinting at superior reproducibility for blood-based testing in African contexts, where assay performance can falter with local sera [10]. Recent systematic reviews echo this, noting HIVST’s safety and effectiveness in sub-Saharan Africa, with delivery models boosting uptake among hard-to-reach groups [13]. For instance, a study in Cameroon on oral HIVST distributed 42,687 kits, yielding 85% outcome reporting and 2.3% positivity, highlighting self-testing’s role in scaling diagnoses [14]. In tandem, the ATLAS program’s modeling in West Africa projects substantial reductions in infections and deaths through HIVST distribution, aligning with our findings’ implications for population-level impact [15].

User experiences add vivid color to these metrics, revealing a kit that’s intuitive and empowering. Participants rated usability highly, with 94.7% handling components effortlessly and 83.3% collecting samples smoothly, while 76.6% navigated procedures without hitches. Qualitative voices bring this to life: *“I would accept to carry out this test again even without any help, the test is very good, the bands are so clear,”* one user shared, capturing the clarity that fueled 96.5% confidence in results. Challenges surfaced too, clustered around instructional clarity, some users grappled with English-only guides, suggesting visual aids or multilingual versions could minimize the 1.8% who misinterpreted outcomes. Logistic regression pinpointed confusion and errors as key predictors of misreads, emphasizing intuitive design’s role in reducing cognitive load (as per chi-square p = 0.032). Looking across age groups, younger participants (youths) demonstrated the highest independent performance, while older adults, despite reporting more difficulty with instructions, maintained the highest confidence and recommendation rates, suggesting that usability support could be tailored differently across age groups. These insights resonate with a 2024 review on young people in sub-Saharan Africa, where HIVST acceptability was high but use often dipped below 50% due to barriers like anxiety or literacy gaps [16].

Acceptability metrics further bolster the case, with 98.0% willing to reuse and 99.0% ready to recommend, backed by strong Likert scores on trust (mean 4.12, SD = 0.88) and low emotional distress during waits (means 3.57–3.59). Feedback highlighted preferences for shorter result times to curb anxiety, aligning with broader calls for user-centered innovations in HIVST. These acceptability findings were echoed in the feasibility assessment, reinforcing that most users were satisfied with the test as it is currently designed and implemented.

These outcomes dovetail with Uganda’s 2017 HIV testing policy, which embraced self-testing to overcome stigma, time constraints, and access hurdles for key populations [4]. Scaling ConfiSign could accelerate early detection, ART initiation, and prevention, echoing a Frontiers editorial on HIVST’s power in reaching the unreachable in sub-Saharan Africa [17,18]. A Tanzanian protocol for door-to-door distribution similarly projects boosts in coverage, suggesting our facility-based success could extend community-wide [19]. Strengths here include the mixed-methods design, capturing 2,434 diverse participants and resolving the discordant results with ELISA and PCR, yielding a 6.2% positivity rate that mirrors national trends.

Limitations temper this optimism: English-only questionnaires may have biased responses from non-fluent users, and facility recruitment restricts generalizability to unsupervised or rural scenarios. Uganda’s adult literacy rate stands at approximately 80% as of 2022, higher than the sub-Saharan African average of around 67%, indicating relatively strong foundational reading skills that could mitigate some language barriers. However, English proficiency remains moderate, with a 2024 EF EPI score of 518 (rank 54/116 globally, 5/24 in Africa), suggesting that while many Ugandans are literate in local languages or basic English from school curricula, nuanced comprehension in English might still pose challenges for a subset [20,21]. Broader evaluations, perhaps incorporating digital literacy aids, could address these gaps [16]. Ultimately, this study positions ConfiSign as a vital asset for Uganda’s HIV battle—accurate, user-friendly, and feasible, urging interim refinements like gauze inclusion and C/T labels to unlock its full potential in ending the epidemic. Following the study’s insights, a Swahili lay-user instruction for use has been released, alongside clear Control (C) and Test (T) line indicators to improve accessibility and result interpretation.

## Conclusion

The ConfiSign HIV Self-Test has proven itself a vital force in Uganda’s ongoing battle against HIV, delivering strong diagnostic performance with 100% sensitivity and 99.8% specificity that hold up from lab tests to the gritty reality of local clinics. Nearly 95% of users found it a breeze to handle, and an impressive 98% would use it again, showing it is a tool people trust to overcome the shame and distance that too often keep testing out of reach. Feasibility shines through too, with strong scores across operations, performance, and cost, hinting at a smooth fit into Uganda’s fight to hit those 95-95-95 targets.

A few practical enhancements, such as including gauze in the kits and improving the kit visual instructions by adding the control (C) and test band (T) to the test kit results reading zone, could further improve usability and user experience. With such refinements, the ConfiSign HIV Self‑Test is poised to become an even more effective tool for early detection and timely treatment. Through expanding access and strengthening confidence in self-testing, Uganda can accelerate progress toward epidemic control, bringing the nation closer to a future where HIV is no longer a public health threat by 2030. This vision embodies hope, resilience, and equity, ensuring that every community benefits from the promise of innovation in diagnostics.

## Supporting information

S1 Table Frequencies and proportions of the responses, analyzed. (DOCX)

S2 Table Summary indicating the number of errors made by users. (DOCX)

S3 Table Chi-Square test comparing the confusion by instructions and the identification of the control lines. (DOCX)

S4 Table Interpretation of results. (DOCX)

S5 Table Mean scores of Likert-scale items (V4–V12). (DOCX)

S6 Table Willingness to reuse and recommend the test. (DOCX)

S7 Table Summary of the frequencies and proportions of the feasibility responses. (DOCX)
