# Supplementary material for: Field performance evaluation of the GenBody ConfiSign HIV self-test for blood-based HIV screening in Uganda
Source: PLoS One. 2026 Oct 27;21(10):e0361282. doi: 10.1371/journal.pone.0361282 (PMC13712722; doi:10.1371/journal.pone.0361282)
Supplement: Supplementary file 1 [file pone.0361282.s001.docx]

**S1 Table. Frequencies and proportions of the responses, analyzed**

| **Variable** | **Response** | **Frequency** | **Proportion** |
| --- | --- | --- | --- |
| Read instructions before starting? | Yes | 844 | 99.29 |
|  | No | 6 | 0.71 |
| Confused by instructions? | Yes | 45 | 5.4 |
|  | No | 789 | 94.6 |
| Followed steps in correct order? | Yes | 824 | 98.45 |
|  | No | 13 | 1.55 |
| Opened test kit correctly? | Yes | 845 | 99.76 |
|  | No | 2 | 0.24 |
| Handled components hygienically? | Yes | 834 | 99.76 |
|  | No | 2 | 0.24 |
| Used correct sample type? | Yes | 834 | 99.76 |
|  | No | 2 | 0.24 |
| Correctly used lancet? | Yes | 847 | 99.88 |
|  | No | 1 | 0.12 |
| Collected adequate blood sample? | Yes | 836 | 99.76 |
|  | No | 2 | 0.24 |
| Transferred sample correctly? | Yes | 839 | 100 |
|  | No | 0 | 0 |
| Applied correct amount of buffer? | Yes | 841 | 100 |
|  | No | 0 | 0 |
| Waited correct time before reading? | Yes | 847 | 99.88 |
|  | No | 1 | 0.12 |
| Left test undisturbed? | Yes | 838 | 99.76 |
|  | No | 2 | 0.24 |
| Identified control/test lines? | Yes | 840 | 99.64 |
|  | No | 3 | 0.36 |
| Correctly interpreted result? | Yes | 832 | 98.7 |
|  | No | 11 | 1.3 |
| Checked results within valid time? | Yes | 813 | 97.37 |
|  | No | 22 | 2.63 |
| Expressed difficulty at any step? | Yes | 92 | 10.87 |
|  | No | 754 | 89.13 |
| Required external assistance? | Yes | 158 | 18.76 |
|  | No | 684 | 81.24 |
| Test failures or invalid results? | Yes | 11 | 1.33 |
|  | No | 817 | 98.67 |
