# Supplementary material for: Field performance evaluation of the GenBody ConfiSign HIV self-test for blood-based HIV screening in Uganda
Source: PLoS One. 2026 Oct 27;21(10):e0361282. doi: 10.1371/journal.pone.0361282 (PMC13712722; doi:10.1371/journal.pone.0361282)
Supplement: Supplementary file 2 [file pone.0361282.s002.docx]

**S2 Table. Summary indicating the number of errors made by users**

| **Number of errors** | **Frequency** | **Percentage** |
| --- | --- | --- |
| No errors | 784 | 91.7 |
| 1 | 64 | 7.49 |
| 2 | 5 | 0.58 |
| 3 | 2 | 0.23 |
