# Supplementary material for: Field performance evaluation of the GenBody ConfiSign HIV self-test for blood-based HIV screening in Uganda
Source: PLoS One. 2026 Oct 27;21(10):e0361282. doi: 10.1371/journal.pone.0361282 (PMC13712722; doi:10.1371/journal.pone.0361282)
Supplement: Supplementary file 3 [file pone.0361282.s003.docx]

**S3 Table. Chi-Square test comparing the confusion by instructions and the identification of the control lines**

| **Confused by instructions?** | **Identified control/test lines?** | |
| --- | --- | --- |
|  | Yes No | Total |
| Yes | 44 1 | 45 |
| No | 786 2 | 788 |
| Total | 830 3 | 833 |
| Pearson chi2(1) = 4.5964 P = 0.032 | | |
