# Supplementary material for: Field performance evaluation of the GenBody ConfiSign HIV self-test for blood-based HIV screening in Uganda
Source: PLoS One. 2026 Oct 27;21(10):e0361282. doi: 10.1371/journal.pone.0361282 (PMC13712722; doi:10.1371/journal.pone.0361282)
Supplement: Supplementary file 4 [file pone.0361282.s004.docx]

**S4 Table. Interpretation of results**

| **Interpretation of results** | **Frequency** | **Proportions** |
| --- | --- | --- |
| Failed to correctly interpret the result | 15 | 1.75 |
| Correct interpretation of results | 840 | 98.25 |
