# Supplementary material for: Field performance evaluation of the GenBody ConfiSign HIV self-test for blood-based HIV screening in Uganda
Source: PLoS One. 2026 Oct 27;21(10):e0361282. doi: 10.1371/journal.pone.0361282 (PMC13712722; doi:10.1371/journal.pone.0361282)
Supplement: Supplementary file 5 [file pone.0361282.s005.docx]

**S5 Table. Mean scores of Likert-scale items (V4–V12)**

| **Variable** | **Observed** | **Mean** | **Standard Deviation** | **Minimum** | **Maximum** |
| --- | --- | --- | --- | --- | --- |
| V4 | 810 | 4.002469 | 0.965406 | 1 | 5 |
| V5 | 809 | 4.030902 | 0.8873614 | 1 | 5 |
| V6 | 808 | 3.928218 | 0.8118062 | 1 | 5 |
| V7 | 808 | 4.054455 | 0.881873 | 1 | 5 |
| V8 | 805 | 4.170186 | 0.7659453 | 1 | 5 |
| V9 | 809 | 4.124845 | 0.877659 | 1 | 5 |
| V11 | 805 | 3.565217 | 1.116267 | 1 | 5 |
| V12 | 807 | 3.589839 | 1.088649 | 1 | 5 |
