# Supplementary material for: Field performance evaluation of the GenBody ConfiSign HIV self-test for blood-based HIV screening in Uganda
Source: PLoS One. 2026 Oct 27;21(10):e0361282. doi: 10.1371/journal.pone.0361282 (PMC13712722; doi:10.1371/journal.pone.0361282)
Supplement: Supplementary file 6 [file pone.0361282.s006.docx]

**S6 Table. Willingness to reuse and recommend the test**

| Would use this test again | | |
| --- | --- | --- |
|  | Frequency | Percentage |
|  |  |  |
| Yes | 792 | 98.02 |
| No | 16 | 1.98 |
| Total | 808 | 100 |
| Would recommend this test to others | | |
|  | Frequency | Percentage |
| Yes | 799 | 99.01 |
| No | 8 | 0.99 |
| Total | 807 | 100 |
