# Supplementary material for: Field performance evaluation of the GenBody ConfiSign HIV self-test for blood-based HIV screening in Uganda
Source: PLoS One. 2026 Oct 27;21(10):e0361282. doi: 10.1371/journal.pone.0361282 (PMC13712722; doi:10.1371/journal.pone.0361282)
Supplement: Supplementary file 7 [file pone.0361282.s007.docx]

**S7 Table. Summary of the frequencies and proportions of the feasibility responses**

| **Test usable in intended setting** | **Frequencies** | **Proportions** |
| --- | --- | --- |
| Yes | 90 | 100 |
| No | 0 | 0 |
| **Storage conditions manageable** |  |  |
| Yes | 90 | 100 |
| No | 0 | 0 |
| **Components easy to easy to handle** |  |  |
| Yes | 90 | 100 |
| No | 0 | 0 |
| **Waste disposal compliant** |  |  |
| Yes | 90 | 100 |
| No | 0 | 0 |
| **Ease of use** |  |  |
| Very easy | 88 | 97.78 |
| Somewhat easy | 2 | 2.22 |
| **Required assistance** |  |  |
| Yes | 48 | 53.93 |
| No | 41 | 46.07 |
| **Errors in sample or interpretation** |  |  |
| No errors | 86 | 97.3 |
| Some errors | 2 | 2.27 |
| **Test duration** |  |  |
| Under 5min | 15 | 17.24 |
| 5–10min | 11 | 12.64 |
| 10–15min | 53 | 60.9 |
| Over 15min | 8 | 9.2 |
| **Results reliable** |  |  |
| Yes | 86 | 97.73 |
| No | 2 | 2.27 |
| **Test affordable** |  |  |
| Yes | 85 | 98.84 |
| No | 1 | 1.16 |
| **Additional resources needed** |  |  |
| Yes | 6 | 6.9 |
| No | 81 | 93.1 |
| **Integration into programs** |  |  |
| Yes | 85 | 98.84 |
| No | 1 | 1.16 |
| **Users trust results** |  |  |
| Yes | 85 | 98.84 |
| No | 1 | 1.16 |
| **Cultural / social barriers** |  |  |
| No | 4 | 4.65 |
| Yes | 82 | 95.35 |
| **Willing to reuse** |  |  |
| Yes | 82 | 97.62 |
| No | 2 | 2.38 |
| **Health worker recommendation** |  |  |
| Yes | 83 | 98.81 |
| No | 1 | 1.19 |
