# Supplementary material for: Field performance evaluation of the GenBody ConfiSign HIV self-test for blood-based HIV screening in Uganda
Source: PLoS One. 2026 Oct 27;21(10):e0361282. doi: 10.1371/journal.pone.0361282 (PMC13712722; doi:10.1371/journal.pone.0361282)
Supplement: Supplementary file 9 [file pone.0361282.s010.docx]

**RESPONSE TO REVIEWERS**

*Field Performance Evaluation of the GenBody ConfiSign HIV Self-Test for Blood-Based HIV Screening in Uganda*

Manuscript ID: PONE-D-25-68642R1

30th September 2026

Dear Dr. Domfeh and Reviewers,

Thank you for your encouraging assessment of our revised manuscript. We are pleased that both reviewers confirmed the manuscript is technically sound, the statistical analysis is appropriate, the underlying data are fully available, and the writing is clear. Below we reproduce every comment in full, in the order it was received, followed by our response and a description of the corresponding change in the manuscript. Nothing has been omitted or paraphrased from the original comments.

**A. Journal Requirements**

**Comment:** *If the reviewer comments include a recommendation to cite specific previously published works, please review and evaluate these publications to determine whether they are relevant and should be cited. There is no requirement to cite these works unless the editor has indicated otherwise. Please review your reference list to ensure that it is complete and correct. If you have cited papers that have been retracted, please include the rationale for doing so in the manuscript text, or remove these references and replace them with relevant current references. Any changes to the reference list should be mentioned in the rebuttal letter that accompanies your revised manuscript.*

**Author Response:** Neither reviewer recommended additional citations this round. We have reviewed our reference list and confirm it is complete and correct, and that none of the cited works have been retracted; no changes to the reference list were needed.

**Comment:** *As the corresponding author, your ORCID iD is verified in the submission system and will appear in the published article. PLOS supports the use of ORCID, and we encourage all coauthors to register for an ORCID iD and use it as well. Please encourage your coauthors to verify their ORCID iD within the submission system before final acceptance.*

**Author Response:** Noted. The corresponding author's ORCID iD is verified, and we have reminded all co-authors to register and verify their ORCID iDs within the submission system ahead of final acceptance.

**Comment:** *To ensure your figures meet our technical requirements, please review our figure guidelines. You may also use PLOS's free figure tool, NAAS, to help you prepare publication quality figures.*

**Author Response:** Not applicable. The manuscript does not contain any figures, only tables (Tables 1 through 7), so no figure files require review against PLOS's technical specifications.

**B. Reviewer #1**

**Comment 1:** *Table 6: Arrange the age group in order. That is, 'Youth' should come right below the 'Adolescent'.*

**Author Response:** Agreed, and we have made this change. Table 6 now lists the age-group categories in chronological order: Adolescent (14–19), Youths (20–25), Adults (25–59), and Older adults (60+), followed by Missing. This is shown as a tracked change document Line 231 in the revised manuscript.

**Comment 2:** *General comments under results: Results section should mainly report on the 'results' emanating from the work. There are a lot of descriptions/explanations and implications that are not needed in results section. They can be rephrased and inserted to 'Discussion' section, where necessary.*

**Author Response:** Agreed. We reviewed the Results section line by line and removed language that explained, interpreted, or drew implications rather than reported findings. In Participant Demographics, we removed the sentence framing young adults as a priority population and the sentence explaining why the age and sex distribution matters for interpreting later findings, since these are interpretive rather than descriptive of what was found; a version of the priority-population point has been relocated to the Discussion. We also removed the editorializing phrase “These metrics underscore the test’s high accuracy” ahead of the Table 4 citation, and the phrases “indicating strong test usability” and “indicating the self-test’s effectiveness” from the observer feedback in the Usability subsection, retaining only the reported figures. The standalone paragraph interpreting usability differences across age groups (“These findings suggest that while usability is broadly strong across age groups…”) has been removed from Results and a rephrased version now appears in the Discussion, alongside the related material on usability by age. Similarly, the sentence linking the feasibility feedback to the structured acceptability survey has been moved from Results into the Discussion’s acceptability paragraph. These changes are shown as tracked changes throughout the revised manuscript, and the Results section now focuses on reporting findings, with interpretation and implications consolidated in the Discussion.

**C. Reviewer #2**

**Comment:** *(No Response) — all questions above were answered “Yes” or “All comments have been addressed,” with no further comments to the author.*

**Author Response:** We thank Reviewer #2 for confirming that all previous comments have been addressed and for the positive assessment of the manuscript's technical soundness, statistical analysis, data availability, and clarity of writing.

We believe these revisions fully address the remaining points raised by the Academic Editor and Reviewer #1, and we thank the editorial team and both reviewers again for their careful reading and guidance throughout this process. We look forward to your response.

Sincerely,

Judith Kyokushaba

On behalf of the authorship team

30th September 2026
