# Supplementary material for: Field performance evaluation of the GenBody ConfiSign HIV self-test for blood-based HIV screening in Uganda
Source: PLoS One. 2026 Oct 27;21(10):e0361282. doi: 10.1371/journal.pone.0361282 (PMC13712722; doi:10.1371/journal.pone.0361282)
Supplement: Peer review history

# Peer review: Decision Letter 0

**DOI:** 10.1371/journal.pone.0361282.r001

**Authors:** Seth Agyei Domfeh

24 Jun 2026

Dear Dr. Kyokushaba,

Thank you for submitting your manuscript to PLOS ONE. After careful consideration, we feel that it has merit but does not fully meet PLOS ONE’s publication criteria as it currently stands. Therefore, we invite you to submit a revised version of the manuscript that addresses the points raised during the review process.

Please submit your revised manuscript by Aug 08 2026 11:59PM. If you will need more time than this to complete your revisions, please reply to this message or contact the journal office at plosone@plos.org. When you're ready to submit your revision, log on to https://www.editorialmanager.com/pone/ and select the 'Submissions Needing Revision' folder to locate your manuscript file.

- A letter that responds to each point raised by the academic editor and reviewer(s). You should upload this letter as a separate file labeled 'Response to Reviewers'.
- A marked-up copy of your manuscript that highlights changes made to the original version. You should upload this as a separate file labeled 'Revised Manuscript with Track Changes'.
- An unmarked version of your revised paper without tracked changes. You should upload this as a separate file labeled 'Manuscript'.

If you would like to make changes to your financial disclosure, please include your updated statement in your cover letter. Guidelines for resubmitting your figure files are available below the reviewer comments at the end of this letter.

If applicable, we recommend that you deposit your laboratory protocols in protocols.io to enhance the reproducibility of your results. Protocols.io assigns your protocol its own identifier (DOI) so that it can be cited independently in the future. For instructions see: https://journals.plos.org/plosone/s/submission-guidelines#loc-laboratory-protocols. Additionally, PLOS ONE offers an option for publishing peer-reviewed Lab Protocol articles, which describe protocols hosted on protocols.io. Read more information on sharing protocols at https://plos.org/protocols?utm_medium=editorial-email&utm_source=authorletters&utm_campaign=protocols.

As the corresponding author, your ORCID iD is verified in the submission system and will appear in the published article. PLOS supports the use of ORCID, and we encourage all coauthors to register for an ORCID iD and use it as well. Please encourage your coauthors to verify their ORCID iD within the submission system before final acceptance, as unverified ORCID iDs will not appear in the published article. *Only*  the individual author can complete the verification step; PLOS staff *cannot*  verify ORCID iDs on behalf of authors.

We look forward to receiving your revised manuscript.

Kind regards,

Seth Agyei Domfeh, PhD

Academic Editor

PLOS One

Journal Requirements:

When submitting your revision, we need you to address these additional requirements.

1.Please ensure that your manuscript meets PLOS ONE's style requirements, including those for file naming. The PLOS ONE style templates can be found at

https://journals.plos.org/plosone/s/file?id=wjVg/PLOSOne_formatting_sample_main_body.pdf and

https://journals.plos.org/plosone/s/file?id=ba62/PLOSOne_formatting_sample_title_authors_affiliations.pdf

2. We note that the grant information you provided in the ‘Funding Information’ and ‘Financial Disclosure’ sections do not match.

When you resubmit, please ensure that you provide the correct grant numbers for the awards you received for your study in the ‘Funding Information’ section.

3. Thank you for stating the following financial disclosure:

“This study was made possible due to the generous funding and unwavering support from the Uganda Ministry of Health, through the National Health Laboratory and Diagnostic Services (NHLDS), the Aids Control Program (ACP), Alpha Global Science (U) Ltd.”

Please state what role the funders took in the study.  If the funders had no role, please state: "The funders had no role in study design, data collection and analysis, decision to publish, or preparation of the manuscript."

If this statement is not correct you must amend it as needed.

Please include this amended Role of Funder statement in your cover letter; we will change the online submission form on your behalf.

4. We note that there is identifying data in the Supporting Information file “Confisign study electronic data capture tool.xlsx” Due to the inclusion of these potentially identifying data, we have removed this file from your file inventory. Prior to sharing human research participant data, authors should consult with an ethics committee to ensure data are shared in accordance with participant consent and all applicable local laws.

Data sharing should never compromise participant privacy. It is therefore not appropriate to publicly share personally identifiable data on human research participants. The following are examples of data that should not be shared:

-Name, initials, physical address

-Ages more specific than whole numbers

-Internet protocol (IP) address

-Specific dates (birth dates, death dates, examination dates, etc.)

-Contact information such as phone number or email address

-Location data

-ID numbers that seem specific (long numbers, include initials, titled “Hospital ID”) rather than random (small numbers in numerical order)

Data that are not directly identifying may also be inappropriate to share, as in combination they can become identifying. For example, data collected from a small group of participants, vulnerable populations, or private groups should not be shared if they involve indirect identifiers (such as sex, ethnicity, location, etc.) that may risk the identification of study participants

Additional guidance on preparing raw data for publication can be found in our Data Policy (https://journals.plos.org/plosone/s/data-availability#loc-human-research-participant-data-and-other-sensitive-data) and in the following article: http://www.bmj.com/content/340/bmj.c181.long.

Please remove or anonymize all personal information (<specific identifying information in file to be removed>), ensure that the data shared are in accordance with participant consent, and re-upload a fully anonymized data set. Please note that spreadsheet columns with personal information must be removed and not hidden as all hidden columns will appear in the published file.

5. Thank you for providing your underlying data as Supporting Information.

We note that the data set contains text or data that is not in English. Please note that PLOS is an English-language publisher, so we require data sets to be provided in English as well. Please upload an English-language version of your data set.

This will also allow us to determine if your data follows PLOS standards per our Data Availability policy here: https://journals.plos.org/plosone/s/data-availability.

6. Your ethics statement should only appear in the Methods section of your manuscript. If your ethics statement is written in any section besides the Methods, please move it to the Methods section and delete it from any other section. Please ensure that your ethics statement is included in your manuscript, as the ethics statement entered into the online submission form will not be published alongside your manuscript.

7. If the reviewer comments include a recommendation to cite specific previously published works, please review and evaluate these publications to determine whether they are relevant and should be cited. There is no requirement to cite these works unless the editor has indicated otherwise.

[Note: HTML markup is below. Please do not edit.]

Reviewer's Responses to Questions

**Comments to the Author**

1. Is the manuscript technically sound, and do the data support the conclusions?

Reviewer #1: No

Reviewer #2: Yes

**********

2. Has the statistical analysis been performed appropriately and rigorously?

Reviewer #1: No

Reviewer #2: Yes

**********

3. Have the authors made all data underlying the findings in their manuscript fully available?

The PLOS Data policy

Reviewer #1: No

Reviewer #2: Yes

**********

4. Is the manuscript presented in an intelligible fashion and written in standard English?

Reviewer #1: Yes

Reviewer #2: Yes

**********

Reviewer #1: Methods

Make table 1 short by grouping facilities within the same region, and separate them with a comma or semi-colon. No need repeating the names of the regions.

Under study design, you mentioned that the study was conducted from Apr 2025 to May 2025 (line 78), which I believe it’s 2 months. However, under Procedures you mentioned that patient recruitment alone took over 4 months (line 105). Kindly clarify.

Did you test for cross-reactivity? Data on cross-reactivity is very important.

Results

Can you refine the statement under Participant Demographics. Did the authors just collect age and gender under the demographics? This seems scanty. I suggest you obtain a table for participant demographics, and show more data. Just one age category is not enough.

Line 217: What is the meaning of ‘Error! Reference source not found’?

Table 4: How did you arrive at these figures for sensitivity etc? I suggest the authors build a proper table/matrix showing True positives, True negatives, False positives and false negatives, after that, then calculate the metrics.

Line 230-235: Be consistent with the percentages. I suggest you run them to one decimal place. Effect the correction throughout the manuscript.

The conclusion made mention of reliability. How did you test for reliability of the diagnostic kit? This study clearly did not test for reliability. Just sensitivity, specificity, PPV and NPV cannot make you draw this conclusion. To accurately test for reliability, further tests are needed such as likelihood ratios, inter-rater and intra-rater reliability or even an ROC curve.

Reviewer #2: The manuscript is technically sound and addresses key ways to detect HIV in an easy and fast way. The data provided also supports the conclusions. The statistical analysis was performed appropriately, and the authors made all data available in the manuscript.

The authors only gave a few details about the demographics of the participants, stating only the total participants, majority age group (18-29) and sex in line 206.

I think a thorough detail of participants' demographics can make it more appreciable to relate to some points that were raised in Table 6. A few of the participants had difficulties with the kit, and this may be attributed to their age. In line 256 concerns were raised on the inability of some to read and understand English, which I think can be attributed to age. It would be ideal for the authors to show the full demographics in a table to give us the age ranges of all participants and to clarify if they can write a statement to indicate if those who had difficulties using the kit were old or young people who could not read and understand.

**********

what does this mean?). If published, this will include your full peer review and any attached files.

If you choose “no”, your identity will remain anonymous but your review may still be made public.

**Do you want your identity to be public for this peer review?**  For information about this choice, including consent withdrawal, please see our Privacy Policy

Reviewer #1: No

Reviewer #2: No

**********

[NOTE: If reviewer comments were submitted as an attachment file, they will be attached to this email and accessible via the submission site. Please log into your account, locate the manuscript record, and check for the action link "View Attachments". If this link does not appear, there are no attachment files.]

To ensure your figures meet our technical requirements, please review our figure guidelines: https://journals.plos.org/plosone/s/figures

You may also use PLOS’s free figure tool, NAAS, to help you prepare publication quality figures: https://journals.plos.org/plosone/s/figures#loc-tools-for-figure-preparation.

NAAS will assess whether your figures meet our technical requirements by comparing each figure against our figure specifications.

# Author comment: Author response to Decision Letter 1

**DOI:** 10.1371/journal.pone.0361282.r002

12 Sep 2026

RESPONSE TO REVIEWERS

Field Performance Evaluation of the GenBody ConfiSign HIV Self-Test for Blood-Based HIV Screening in Uganda

10th September 2026

Dear Editor and Reviewers,

Thank you for the opportunity to revise our manuscript. We are grateful to the Academic Editor and both reviewers for their careful reading and constructive feedback. Below we reproduce every comment in full, in the order it was received, followed by our response and a description of the corresponding change in the manuscript. Nothing has been omitted or paraphrased from the original comments.

A. PLOS ONE Editorial and Journal Requirements

Comment 1: Please ensure that your manuscript meets PLOS ONE's style requirements, including those for file naming. The PLOS ONE style templates can be found at

Author Response: We removed subtitles from the abstract to comply with PLOS ONE's style requirements. The abstract now presents Background, Methods, Results, and Conclusions as continuous unlabeled prose per the journal template, and file names have been checked against PLOS ONE's naming conventions.

Comment: We note that the grant information you provided in the 'Funding Information' and 'Financial Disclosure' sections do not match.

When you resubmit, please ensure that you provide the correct grant numbers for the awards you received for your study in the 'Funding Information' section.

Author Response: This has been clarified. The Funding Information and Financial Disclosure statements have been aligned and now read identically (see our response to Comment 3 below, which reproduces the harmonized statement).

Comment 3: Thank you for stating the following financial disclosure:

“This study was supported by Alpha Global Science (U) Limited and the Ministry of Health, Uganda under grant number MoH 2024_001. Alpha Global Science (U) Limited provided HIV Confisign self-testing kits and technical support, and research funding to support field study teams. The Ministry of Health, Uganda provided in-kind support for all testing-related infrastructure and consumables, including applicable comparator supplies, in addition to technical personnel who led the study. The funders had no role in study design, data collection and analysis, decision to publish, or preparation of the manuscript."

If this statement is not correct, you must amend it as needed.

Please include this amended Role of Funder statement in your cover letter; we will change the online submission form on your behalf.

Author Response: Funding: This study was supported by Alpha Global Science (U) Limited and the Ministry of Health, Uganda under grant number MoH 2024_001. Alpha Global Science (U) Limited provided HIV ConfiSign self-testing kits and technical support, and research funding to support field study teams. The Ministry of Health, Uganda provided in-kind support for all testing-related infrastructure and consumables, including applicable comparator supplies, in addition to technical personnel who led the study. The funders had no role in study design, data collection and analysis, decision to publish, or preparation of the manuscript. This statement is also reflected in our cover letter.

Competing interests: JK and RM contributed to resource mobilization. The other authors have declared that no competing interests exist.

Comment 4: We note that there is identifying data in the Supporting Information file “Confisign study electronic data capture tool.xlsx” Due to the inclusion of these potentially identifying data, we have removed this file from your file inventory. Prior to sharing human research participant data, authors should consult with an ethics committee to ensure data are shared in accordance with participant consent and all applicable local laws.

Data sharing should never compromise participant privacy. It is therefore not appropriate to publicly share personally identifiable data on human research participants. The following are examples of data that should not be shared:

-Name, initials, physical address

-Ages more specific than whole numbers

-Internet protocol (IP) address

-Specific dates (birth dates, death dates, examination dates, etc.)

-Contact information such as phone number or email address

-Location data

-ID numbers that seem specific (long numbers, include initials, titled “Hospital ID”) rather than random (small numbers in numerical order)

Data that are not directly identifying may also be inappropriate to share, as in combination they can become identifying. For example, data collected from a small group of participants, vulnerable populations, or private groups should not be shared if they involve indirect identifiers (such as sex, ethnicity, location, etc.) that may risk the identification of study participants

Additional guidance on preparing raw data for publication can be found in our Data Policy (https://journals.plos.org/plosone/s/data-availability#loc-human-research-participant-data-and-other-sensitive-data) and in the following article: http://www.bmj.com/content/340/bmj.c181.long.

Please remove or anonymize all personal information (<specific identifying information in file to be removed>), ensure that the data shared are in accordance with participant consent, and re-upload a fully anonymized data set. Please note that spreadsheet columns with personal information must be removed and not hidden as all hidden columns will appear in the published file.

Author Response: This has been done. The facility register/patient numbers in every sheet of the electronic data capture tool have been replaced with new Participant IDs generated solely for this dataset (e.g., MSK-001), which cannot be traced back to any facility register. Exact testing dates have been removed from every row, since a specific date combined with a small facility’s daily patient count could risk re-identification; the overall study period (09 April to 30 May 2025) remains reported in the manuscript. No names, initials, addresses, phone numbers, email addresses, IP addresses, or location data were present in the original file, and no hidden rows, columns, or cell comments were found. A README tab documenting these changes has been added to the workbook, and the fully anonymized dataset has been re-uploaded as Supporting Information.

Comment 5: Thank you for providing your underlying data as Supporting Information.

We note that the data set contains text or data that is not in English. Please note that PLOS is an English-language publisher, so we require data sets to be provided in English as well. Please upload an English-language version of your data set.

This will also allow us to determine if your data follows PLOS standards per our Data Availability policy here: https://journals.plos.org/plosone/s/data-availability.

Author Response: We have reviewed and only uploaded the English consent and assent forms (stamped).

Comment 6: Your ethics statement should only appear in the Methods section of your manuscript. If your ethics statement is written in any section besides the Methods, please move it to the Methods section and delete it from any other section. Please ensure that your ethics statement is included in your manuscript, as the ethics statement entered into the online submission form will not be published alongside your manuscript.

Author Response: We reviewed the manuscript and streamlined the ethics-related content into a single "Ethical Considerations" subsection within the Methods, removing the separate "Risk Management and Reporting" and "Confidentiality and Data Protection" headings and folding their essential content into that one subsection so nothing is repeated elsewhere in the manuscript. The corresponding text (previously spanning lines 196-206) has been deleted and condensed to reflect the confidentiality aspects of the study within the Ethical Considerations subsection.

Comment 7: If the reviewer comments include a recommendation to cite specific previously published works, please review and evaluate these publications to determine whether they are relevant and should be cited. There is no requirement to cite these works unless the editor has indicated otherwise.

Author Response: NA – no specific previously published works were recommended for citation by the reviewers.

B. Reviewer #1

Reviewer #1 introduced their comments as follows: "Please use the space provided to explain your answers to the questions above. You may also include additional comments for the author, including concerns about dual publication, research ethics, or publication ethics." The reviewer’s comments were organized under two headings, "Methods" and "Results," and are addressed point by point below in the order received.

Methods

Comment 1: Make table 1 short by grouping facilities within the same region, and separate them with a comma or semi-colon. No need repeating the names of the regions.

Author Response: We agree, and have amended this. Table 1 has been reformatted so each region appears once, with its facilities listed together and separated by semi-colons (e.g., "Western: Mbarara Municipal HCIV; Kibito HC IV; Fort Portal Regional Referral Hospital; Kabale Regional Referral Hospital"), removing the previous repetition of region names across multiple rows. This change is shown as a tracked change in the revised manuscript.

Comment 2: Under study design, you mentioned that the study was conducted from Apr 2025 to May 2025 (line 78), which I believe it's 2 months. However, under Procedures you mentioned that patient recruitment alone took over 4 months (line 105). Kindly clarify.

Author Response: Thank you for catching this inconsistency. We have changed line 105 to correctly state that patient recruitment was conducted within 52 days (09 April to 30 May 2025), consistent with the overall study period stated under Study Design. The earlier reference to "four months" was an error and has been corrected as a tracked change.

Comment 3: Did you test for cross-reactivity? Data on cross-reactivity is very important.

Author Response: We did not independently test for cross-reactivity in this field evaluation, because the manufacturer’s validation report had already indicated no cross-reactivity to antibodies against common pathogens. We have added this clarification to the manuscript so readers understand the basis for not repeating cross-reactivity testing in the field.

Results

Comment 4: Can you refine the statement under Participant Demographics? Did the authors just collect age and gender under the demographics? This seems scanty. I suggest you obtain a table for participant demographics and show more data. Just one age category is not enough.

Author Response: We have refined and expanded the Participant Demographics section in response to this comment. Specifically, the age distribution has now been expanded to cover all age categories represented in the study population, rather than reporting only the majority age group. In addition, we expanded the demographic analysis among the 798 participants who underwent qualitative analysis to include sex, age, and education level; we did not capture any other demographic data beyond these in the study tool and therefore could not be added retrospectively. The revised manuscript now presents the available demographic characteristics in a more complete table beginning at line 273, allowing readers to better contextualize the usability and acceptability findings discussed later in the Results.

Comment 5: Line 217: What is the meaning of 'Error! Reference source not found'?

Author Response: This was a broken cross-reference field that failed to render correctly when the document was last saved. We have removed this error from the report and verified that all table and figure references now resolve and display correctly throughout the manuscript.

Comment 6: Table 4: How did you arrive at these figures for sensitivity, etc? I suggest the authors build a proper table/matrix showing True positives, True negatives, False positives, and false negatives; after that, calculate the metrics.

Author Response: We agree, and have built the requested 2×2 matrix directly into the manuscript as Table 4 (True Positives, False Positives, False Negatives, True Negatives), from which sensitivity, specificity, PPV, and NPV are calculated. The underlying raw data are also provided as Supporting Information for full traceability.

Comment 7: Line 230-235: Be consistent with the percentages. I suggest you run them to one decimal place. Effect the correction throughout the manuscript.

Author Response: We agree and have standardized every percentage in the manuscript to one decimal place for consistency, including the usability and acceptability figures previously reported to two decimal places (e.g., 94.74% → 94.7%; 83.33% → 83.3%; 95.11% → 95.1%; 95.24% → 95.2%; 98.02% → 98.0%; 99.01% → 99.0%). These are marked as tracked changes throughout the revised manuscript.

Comment 8: The conclusion made mention of reliability. How did you test for the reliability of the diagnostic kit? This study clearly did not test for reliability. Just sensitivity, specificity, PPV and NPV cannot make you draw this conclusion. To accurately test for reliability, further tests are needed such as likelihood ratios, inter-rater and intra-rater reliability or even an ROC curve.

Author Response: This is a fair and important correction. We have removed the term "reliability" from the abstract conclusion and from the Conclusion section, since this study measured diagnostic accuracy (sensitivity, specificity, PPV, NPV) and not reliability in the psychometric or test-retest sense. The revised text now refers to the kit’s "diagnostic accuracy" and "strong diagnostic performance" instead, which accurately reflects what was measured. We agree that establishing reliability would require likelihood ratios, inter-rater and intra-rater agreement, or ROC analysis, which were outside the scope of this field evaluation and have not been claimed.

C. Reviewer #2

Comment 1 (overall assessment): The manuscript is technically sound and addresses key ways to detect HIV in an easy and fast way. The data provided also supports the conclusions. The statistical analysis was performed appropriately, and the authors made all data available in the manuscript.

Author Response: We thank the reviewer for this positive assessment of the manuscript’s technical soundness, conclusions, statistical analysis, and data availability.

Comment 2 (participant demographics): The authors only gave a few details about the demographics of the participants, stating only the total participants, majority age group (18-29) and sex in line 206. I think a thorough detail of participants' demographics can make it more appreciable to relate to some points that were raised in Table 6. A few of the participants had difficulties with the kit, and this may be attributed to their age. In line 256 concerns were raised on the inability of some to read and understand English, which I think can be attributed to age. It would be ideal for the authors to show the full demographics in a table to give us the age ranges of all participants and to clarify if they can write a statement to indicate if those who had difficulties using the kit were old or young people who could not read and understand.

Author Response: Agreed, and we have made these changes. We have expanded the Participant Demographics section into a comprehensive table showing the full age range of all participants rather than only the majority age group. In the Results discussion of usability challenges (relating to Table 7), we have added a statement clarifying the association between age, English proficiency, and the difficulties some participants experienced interpreting the test results, so readers can better judge whether these difficulties were concentrated among older or younger participants and among those with limited English comprehension.

Similar to the response to comment 4, we have refined and expanded the Participant Demographics section in response to this comment. The age distribution has now been expanded to cover all age categories represented in the study population, rather than reporting only the majority age group. In addition, we expanded the demographic analysis among the 798 participants who underwent qualitative analysis to include sex, age, and education level; we also added further analysis to show how the usability challenges fared among different age groups.

D. New Changes From the Authors

In addition to the point-by-point revisions above, the author team is reporting the following change, made independently of reviewer comments:

• Caroline Makoha (CM) and Flaviano Mugalula (FM) have been added as co-authors in recognition of their contributions to data curation and analysis.

E. PLOS ONE Follow-Up on Resubmission

Following our last resubmission, PLOS ONE returned the manuscript with three further items to address before we could proceed. Each is reproduced in full below, followed by our response.

Comment 1: We note that the grant information you provided in the ‘Funding Information’ and ‘Financial Disclosure’ sections do not match. When you resubmit, please ensure that you provide the correct grant numbers for the awards you received for your study in the ‘Funding Information’ section.

Author Response: On review, we found that the grant reference MoH 2024_001, part of the Financial Disclosure statement PLOS quoted back to us earlier in this process, had been dropped when we harmonized the funding statement during our last revision, which is why the Funding Information and Financial Disclosure sections no longer matched. We have restored the grant number throughout: the Financial Disclosure statement (see our response to Comment 3 in Section A above) now reads “...under grant number MoH 2024_001”, and the same number, MoH 2024_001, is reflected in the Funding Information section of the online submission form and in our cover letter, so all three now match.

Comment 2: Please ensure that you refer to Figure 7 in your text as, if accepted, production will need this reference to link the reader to the figure.

Author Response: The manuscript does not contain any figures, only tables (Tables 1 through 7), so we believe this refers to Table 7 (Distribution of Participant Responses on Usability, Comprehension, and Acceptability). On review we found that, unlike Tables 1 through 6, Table 7 was not explicitly cited by name in the text before the table appeared, only its caption preceded it. We have corrected this: the sentence immediately before the table now reads “...and 95.2% would recommend it to other clients (Table 7).”, giving production the explicit in-text reference requested.

Comment 3: We note that your author list was updated during the revision process. In order to add or remove authors or update the order of the author byline after initial submission, we ask that authors complete an Authorship Change Request form. You may review our full authorship change policy and download the Authorship Change Request form here: https://journals.plos.org/plosone/s/authorship#loc-authorship-changes. Please fill out all required sections of the form. If you are adding or removing more than 2 authors, you can complete multiple forms and submit as many forms as needed to reflect the updates. Please return the form(s) as an attachment by emailing plosone@plos.org or by uploading it as a submission file labeled with the file type ‘Other’. The form will be reviewed by PLOS staff for approval. Should the changes be approved, we will require written confirmation from all authors confirming that they approve of the changes. Please note that if your manuscript is accepted, we will not be able to complete the publication process without the completed form.

Author Response: Noted. As described in Section D above, Caroline Makoha and Flaviano Mugalula have been added as co-authors in recognition of their contributions to data curation and analysis, and the author byline order has been updated to move Dennis Mujuni and Viola Kasone into more prominent positions, reflecting their substantially expanded roles during the revision process; this is now reflected in the revised manuscript. We are completing the Authorship Change Request form to document both changes, including the reason for each addition and the reason for the reordering, and uploading it as a submission file under the file type ‘Other’, as instructed.

We believe these revisions fully address the concerns raised by the Academic Editor and both reviewers, and we thank you again for the opportunity to strengthen the manuscript. We look forward to your response.

Sincerely,

Judith Kyokushaba

On behalf of the authorship team

10th September 2026

Attachment Submitted filename: Response to Reviewers.docx

# Peer review: Decision Letter 1

**DOI:** 10.1371/journal.pone.0361282.r003

**Authors:** Seth Agyei Domfeh

29 Sep 2026

Dear Dr. Kyokushaba,

Thank you for submitting your manuscript to PLOS One. After careful consideration, we feel that it has merit but does not fully meet PLOS One’s publication criteria as it currently stands. Therefore, we invite you to submit a revised version of the manuscript that addresses the points raised during the review process.

Please submit your revised manuscript by Nov 13 2026 11:59PM. If you will need more time than this to complete your revisions, please reply to this message or contact the journal office at plosone@plos.org. When you're ready to submit your revision, log on to https://www.editorialmanager.com/pone/ and select the 'Submissions Needing Revision' folder to locate your manuscript file.

- A letter that responds to each point raised by the academic editor and reviewer(s). You should upload this letter as a separate file labeled 'Response to Reviewers'.
- A marked-up copy of your manuscript that highlights changes made to the original version. You should upload this as a separate file labeled 'Revised Manuscript with Track Changes'.
- An unmarked version of your revised paper without tracked changes. You should upload this as a separate file labeled 'Manuscript'.

If you would like to make changes to your financial disclosure, please include your updated statement in your cover letter. Guidelines for resubmitting your figure files are available below the reviewer comments at the end of this letter.

If applicable, we recommend that you deposit your laboratory protocols in protocols.io to enhance the reproducibility of your results. Protocols.io assigns your protocol its own identifier (DOI) so that it can be cited independently in the future. For instructions see: https://journals.plos.org/plosone/s/submission-guidelines#loc-laboratory-protocols. Additionally, PLOS One offers an option for publishing peer-reviewed Lab Protocol articles, which describe protocols hosted on protocols.io. Read more information on sharing protocols at https://plos.org/protocols?utm_medium=editorial-email&utm_source=authorletters&utm_campaign=protocols.

As the corresponding author, your ORCID iD is verified in the submission system and will appear in the published article. PLOS supports the use of ORCID, and we encourage all coauthors to register for an ORCID iD and use it as well. Please encourage your coauthors to verify their ORCID iD within the submission system before final acceptance, as unverified ORCID iDs will not appear in the published article. *Only*  the individual author can complete the verification step; PLOS staff *cannot*  verify ORCID iDs on behalf of authors.

We look forward to receiving your revised manuscript.

Kind regards,

Seth Agyei Domfeh, PhD

Academic Editor

PLOS One

Journal Requirements:

If the reviewer comments include a recommendation to cite specific previously published works, please review and evaluate these publications to determine whether they are relevant and should be cited. There is no requirement to cite these works unless the editor has indicated otherwise.

Please review your reference list to ensure that it is complete and correct. If you have cited papers that have been retracted, please include the rationale for doing so in the manuscript text, or remove these references and replace them with relevant current references. Any changes to the reference list should be mentioned in the rebuttal letter that accompanies your revised manuscript. If you need to cite a retracted article, indicate the article’s retracted status in the References list and also include a citation and full reference for the retraction notice.

[Note: HTML markup is below. Please do not edit.]

Reviewers' comments:

Reviewer's Responses to Questions

**Comments to the Author**

Reviewer #1: All comments have been addressed

Reviewer #2: All comments have been addressed

**********

2. Is the manuscript technically sound, and do the data support the conclusions?

Reviewer #1: Yes

Reviewer #2: Yes

**********

3. Has the statistical analysis been performed appropriately and rigorously?

Reviewer #1: Yes

Reviewer #2: Yes

**********

4. Have the authors made all data underlying the findings in their manuscript fully available?

The PLOS Data policy

Reviewer #1: Yes

Reviewer #2: Yes

**********

5. Is the manuscript presented in an intelligible fashion and written in standard English?

Reviewer #1: Yes

Reviewer #2: Yes

**********

Reviewer #1: Table 6: Arrange the age group in order. That is, ‘Youth’ should come right below the ‘Adolescent’.

General comments under results: Results section should mainly report on the ‘results’ emanating from the work. There are a lot of descriptions/explanations and implications that are not needed in results section. They can be rephrased and inserted to ‘Discussion’ section, where necessary.

Reviewer #2: (No Response)

**********

what does this mean?). If published, this will include your full peer review and any attached files.

If you choose “no”, your identity will remain anonymous but your review may still be made public.

**Do you want your identity to be public for this peer review?**  For information about this choice, including consent withdrawal, please see our Privacy Policy

Reviewer #1: No

Reviewer #2: No

**********

[NOTE: If reviewer comments were submitted as an attachment file, they will be attached to this email and accessible via the submission site. Please log into your account, locate the manuscript record, and check for the action link "View Attachments". If this link does not appear, there are no attachment files.]

To ensure your figures meet our technical requirements, please review our figure guidelines: https://journals.plos.org/plosone/s/figures

You may also use PLOS’s free figure tool, NAAS, to help you prepare publication quality figures: https://journals.plos.org/plosone/s/figures#loc-tools-for-figure-preparation.

NAAS will assess whether your figures meet our technical requirements by comparing each figure against our figure specifications.

# Author comment: Author response to Decision Letter 2

**DOI:** 10.1371/journal.pone.0361282.r004

6 Oct 2026

RESPONSE TO REVIEWERS

Field Performance Evaluation of the GenBody ConfiSign HIV Self-Test for Blood-Based HIV Screening in Uganda

Manuscript ID: PONE-D-25-68642R1

30th September 2026

Dear Dr. Domfeh and Reviewers,

Thank you for your encouraging assessment of our revised manuscript. We are pleased that both reviewers confirmed the manuscript is technically sound, the statistical analysis is appropriate, the underlying data are fully available, and the writing is clear. Below we reproduce every comment in full, in the order it was received, followed by our response and a description of the corresponding change in the manuscript. Nothing has been omitted or paraphrased from the original comments.

A. Journal Requirements

Comment: If the reviewer comments include a recommendation to cite specific previously published works, please review and evaluate these publications to determine whether they are relevant and should be cited. There is no requirement to cite these works unless the editor has indicated otherwise. Please review your reference list to ensure that it is complete and correct. If you have cited papers that have been retracted, please include the rationale for doing so in the manuscript text, or remove these references and replace them with relevant current references. Any changes to the reference list should be mentioned in the rebuttal letter that accompanies your revised manuscript.

Author Response: Neither reviewer recommended additional citations this round. We have reviewed our reference list and confirm it is complete and correct, and that none of the cited works have been retracted; no changes to the reference list were needed.

Comment: As the corresponding author, your ORCID iD is verified in the submission system and will appear in the published article. PLOS supports the use of ORCID, and we encourage all coauthors to register for an ORCID iD and use it as well. Please encourage your coauthors to verify their ORCID iD within the submission system before final acceptance.

Author Response: Noted. The corresponding author's ORCID iD is verified, and we have reminded all co-authors to register and verify their ORCID iDs within the submission system ahead of final acceptance.

Comment: To ensure your figures meet our technical requirements, please review our figure guidelines. You may also use PLOS's free figure tool, NAAS, to help you prepare publication quality figures.

Author Response: Not applicable. The manuscript does not contain any figures, only tables (Tables 1 through 7), so no figure files require review against PLOS's technical specifications.

B. Reviewer #1

Comment 1: Table 6: Arrange the age group in order. That is, 'Youth' should come right below the 'Adolescent'.

Author Response: Agreed, and we have made this change. Table 6 now lists the age-group categories in chronological order: Adolescent (14–19), Youths (20–25), Adults (25–59), and Older adults (60+), followed by Missing. This is shown as a tracked change document Line 231 in the revised manuscript.

Comment 2: General comments under results: Results section should mainly report on the 'results' emanating from the work. There are a lot of descriptions/explanations and implications that are not needed in results section. They can be rephrased and inserted to 'Discussion' section, where necessary.

Author Response: Agreed. We reviewed the Results section line by line and removed language that explained, interpreted, or drew implications rather than reported findings. In Participant Demographics, we removed the sentence framing young adults as a priority population and the sentence explaining why the age and sex distribution matters for interpreting later findings, since these are interpretive rather than descriptive of what was found; a version of the priority-population point has been relocated to the Discussion. We also removed the editorializing phrase “These metrics underscore the test’s high accuracy” ahead of the Table 4 citation, and the phrases “indicating strong test usability” and “indicating the self-test’s effectiveness” from the observer feedback in the Usability subsection, retaining only the reported figures. The standalone paragraph interpreting usability differences across age groups (“These findings suggest that while usability is broadly strong across age groups…”) has been removed from Results and a rephrased version now appears in the Discussion, alongside the related material on usability by age. Similarly, the sentence linking the feasibility feedback to the structured acceptability survey has been moved from Results into the Discussion’s acceptability paragraph. These changes are shown as tracked changes throughout the revised manuscript, and the Results section now focuses on reporting findings, with interpretation and implications consolidated in the Discussion.

C. Reviewer #2

Comment: (No Response) — all questions above were answered “Yes” or “All comments have been addressed,” with no further comments to the author.

Author Response: We thank Reviewer #2 for confirming that all previous comments have been addressed and for the positive assessment of the manuscript's technical soundness, statistical analysis, data availability, and clarity of writing.

We believe these revisions fully address the remaining points raised by the Academic Editor and Reviewer #1, and we thank the editorial team and both reviewers again for their careful reading and guidance throughout this process. We look forward to your response.

Sincerely,

Judith Kyokushaba

On behalf of the authorship team

30th September 2026

Attachment Submitted filename: Response to Reviewers_R2.docx

# Peer review: Decision Letter 2

**DOI:** 10.1371/journal.pone.0361282.r005

**Authors:** Seth Agyei Domfeh

9 Oct 2026

Field Performance Evaluation of the GenBody ConfiSign HIV Self-Test for Blood-Based HIV Screening in Uganda

PONE-D-25-68642R2

Dear Dr. Kyokushaba,

We’re pleased to inform you that your manuscript has been judged scientifically suitable for publication and will be formally accepted for publication once it meets all outstanding technical requirements.

Within one week, you’ll receive an e-mail detailing the required amendments. When these have been addressed, you’ll receive a formal acceptance letter and your manuscript will be scheduled for publication.

An invoice will be generated when your article is formally accepted. Please note, if your institution has a publishing partnership with PLOS and your article meets the relevant criteria, all or part of your publication costs will be covered. Please make sure your user information is up-to-date by logging into Editorial Manager at Editorial Manager® and clicking the ‘Update My Information' link at the top of the page. For questions related to billing, please contact billing support.

If your institution or institutions have a press office, please notify them about your upcoming paper to help maximize its impact. If they’ll be preparing press materials, please inform our press team as soon as possible -- no later than 48 hours after receiving the formal acceptance. Your manuscript will remain under strict press embargo until 2 pm Eastern Time on the date of publication. For more information, please contact onepress@plos.org.

Kind regards,

Seth Agyei Domfeh, PhD

Academic Editor

PLOS One

Reviewers' comments:

Reviewer's Responses to Questions

**Comments to the Author**

Reviewer #1: All comments have been addressed

**********

2. Is the manuscript technically sound, and do the data support the conclusions?

Reviewer #1: Yes

**********

3. Has the statistical analysis been performed appropriately and rigorously?

Reviewer #1: Yes

**********

4. Have the authors made all data underlying the findings in their manuscript fully available?

The PLOS Data policy

Reviewer #1: Yes

**********

5. Is the manuscript presented in an intelligible fashion and written in standard English?

Reviewer #1: Yes

**********

Reviewer #1: The authors have addressed all concerning remarks in this manuscript, and it is in good shape now. I do not have any further comment(s) at this moment.

**********

what does this mean?). If published, this will include your full peer review and any attached files.

If you choose “no”, your identity will remain anonymous but your review may still be made public.

**Do you want your identity to be public for this peer review?**  For information about this choice, including consent withdrawal, please see our Privacy Policy

Reviewer #1: No

**********

# Editor report: Acceptance letter

**DOI:** 10.1371/journal.pone.0361282.r006

**Authors:** Seth Agyei Domfeh

PONE-D-25-68642R2

PLOS One

Dear Dr. Kyokushaba,

I'm pleased to inform you that your manuscript has been deemed suitable for publication in PLOS One. Congratulations! Your manuscript is now being handed over to our production team.

At this stage, our production department will prepare your paper for publication. This includes ensuring the following:

* All references, tables, and figures are properly cited

* All relevant supporting information is included in the manuscript submission,

* There are no issues that prevent the paper from being properly typeset

You will receive further instructions from the production team, including instructions on how to review your proof when it is ready. Please keep in mind that we are working through a large volume of accepted articles, so please give us a few days to review your paper and let you know the next and final steps.

Lastly, if your institution or institutions have a press office, please let them know about your upcoming paper now to help maximize its impact. If they'll be preparing press materials, please inform our press team within the next 48 hours. Your manuscript will remain under strict press embargo until 2 pm Eastern Time on the date of publication. For more information, please contact onepress@plos.org.

You will receive an invoice from PLOS for your publication fee after your manuscript has reached the completed accept phase. If you receive an email requesting payment before acceptance or for any other service, this may be a phishing scheme. Learn how to identify phishing emails and protect your accounts at https://explore.plos.org/phishing.

If we can help with anything else, please email us at customercare@plos.org.

Thank you for submitting your work to PLOS One and supporting open access.

Kind regards,

PLOS One Editorial Office Staff

on behalf of

Dr. Seth Agyei Domfeh

Academic Editor

PLOS One
